# Effects of Cocamidopropyl Betaine on In Vitro Rumen Fermentation and Enzyme Spatial Distribution, and In Vivo Digestibility and Growth Performance of Growing Yaks

**DOI:** 10.3390/ani16101505

**Published:** 2026-05-14

**Authors:** Mingyu Cao, Lianghao Lu, Chong Shao, Jia Zhou, Xiaolin Wang, Bai Xue

**Affiliations:** 1 Animal Nutrition Institute, Sichuan Agricultural University, Chengdu 611130, China; mycao2021114018@163.com (M.C.); 13551012639@163.com (L.L.); schong0510@163.com (C.S.); wxl547312630@163.com (X.W.); 2 Chongqing Academy of Animal Sciences, Chongqing 402460, China; jjzhouz@163.com

**Keywords:** amphoteric surfactant, apparent digestibility, cocamidopropyl betaine, fibrolytic enzymes, forage utilization, growth performance, in vitro rumen fermentation, rumen microbiology

## Abstract

Yaks (*Bos grunniens*) on the Qinghai–Tibetan Plateau face severe nutritional limitations during the dry season due to dependence on highly lignified, low-quality roughage. Identifying safe and effective rumen regulators capable of enhancing fiber utilization in this species is therefore of great practical importance. This study employed a two-pronged approach integrating in vitro mechanistic investigation and in vivo validation to evaluate the effects of the amphoteric surfactant cocamidopropyl betaine (CAPB) on rumen fermentation, the micro-spatial distribution of digestive enzymes, apparent total tract digestibility, and the macroscopic growth performance of yaks. In the in vitro fermentation trial (Experiment 1), a randomized block design was employed where a straw-based high-forage diet was used as the substrate and supplemented with 0, 0.5, 1.0, 1.5, 2.0, 2.5, and 3.0% CAPB (based on substrate dry matter, DM) for a 48 h batch culture. The results showed that as the CAPB supplementation level increased, cumulative gas production, the degradation rates of DM and neutral detergent fiber (NDF), and the yields of total volatile fatty acids and microbial protein all exhibited significant quadratic responses (*p* < 0.05), peaking at the 0.5–1.0% supplementation levels. Concurrently, CAPB significantly promoted the transfer and release of carboxymethyl cellulase and xylanase into the free liquid phase (*p* < 0.01). In the in vivo validation trial (Experiment 2), 24 healthy growing male yaks (initial body weight 131.2 ± 8.4 kg) were allocated in a completely randomized design to four groups and fed a basal diet supplemented with 0, 0.5, 1.0, or 2.0% CAPB for 44 days. The results indicated that, while maintaining a stable DM intake, the addition of 0.5% CAPB significantly increased the average daily gain (ADG) of yaks (*p* < 0.05), improved the feed-to-gain ratio, and significantly enhanced the apparent total tract digestibility of NDF and ether extract (*p* < 0.05). However, when the supplementation dose exceeded the safety threshold (≥2.5% in vitro and ≥2.0% in vivo), both fermentation parameters and growth advantages declined. In conclusion, under the present experimental conditions, 0.5% CAPB improved roughage fermentation efficiency, putatively through an ‘enzyme elution’ mechanism, and was associated with macroscopic improvements in NDF and EE apparent digestibility and ADG in growing yaks. These findings identify 0.5% CAPB as a promising candidate rumen regulator for improving roughage utilization in growing yaks; broader generalization will require larger-scale and longer-duration trials.

## Simple Summary

Yaks in the alpine pastoral areas of the Qinghai–Tibetan Plateau primarily consume highly lignified, low-quality roughage during the 6-month dry season, leading to low digestive efficiency and growth stagnation. This study explored the use of cocamidopropyl betaine (CAPB), a novel feed additive with both positive and negative charges that allows it to work effectively in the complex rumen environment, to overcome this nutritional bottleneck. Through a dual validation approach combining laboratory simulations and live animal feeding, we found that low-dose supplementation of CAPB significantly improved the ability of rumen fluid to penetrate and wet the surface of forage particles and promoted the detachment and release of digestive enzymes from bacterial surfaces into the free liquid phase of the rumen, thereby enabling a deeper degradation of otherwise hard-to-digest fibers. The results demonstrated that dietary supplementation with 0.5% CAPB significantly enhanced both the daily weight gain and nutrient digestibility of yaks. These findings provide an efficient, safe, and practically applicable novel strategy for improving roughage utilization in ruminants during the dry season in alpine regions.

## 1. Introduction

Yaks (*Bos grunniens*) are a key livestock species endemic to the alpine pastoral areas of the Qinghai–Tibetan Plateau. During the dry season, their diets rely heavily on roughage such as rice straw [1,2]. However, the high degree of lignification and complex physical structure of such feedstuffs restrict the attachment and degradation by rumen microorganisms [3,4]. To promote the utilization of roughage, surfactants (e.g., non-ionic Tween 80) have been introduced into ruminant nutrition research to improve fermentation efficiency by enhancing substrate wettability [5,6,7]. Nevertheless, owing to the lack of molecular charges, traditional non-ionic surfactants exhibit limited binding efficiency to the substrate surface and typically require relatively high supplementation doses to be effective, which often induces cytotoxicity to the rumen microecology [8,9]. Therefore, it is imperative to identify novel alternatives that offer both high surface activity and excellent biocompatibility.

Cocamidopropyl betaine (CAPB) is an amphoteric surfactant that contains both positively and negatively charged groups within its molecular structure [10]. This characteristic enables it to better adapt to the rumen microenvironment and effectively reduce the solid–liquid interfacial tension at low concentrations, thereby avoiding the toxicity risks associated with high dosages [11]. More importantly, studies have demonstrated that CAPB exhibits extremely low biological toxicity, and its unique amphoteric structure can effectively prevent the non-specific adsorption of proteins, significantly enhancing the structural stability of proteins in the aqueous phase [12]. This dual capacity—reducing interfacial tension while simultaneously stabilizing enzyme protein conformation—is of particular relevance to the mechanistic bottleneck that constrains fiber digestion in the rumen. Under natural conditions, fibrolytic bacteria in the rumen primarily rely on “cellulosomes” located on their cell walls to attach to the substrate surface for “contact digestion” [13]. These cellulosomal enzyme complexes are anchored to the bacterial outer membrane through hydrophobic protein–protein interactions, and their activity is therefore physically constrained by the size of the intact bacterial cell [14]. Because of their relatively large size, intact bacterial cells experience significant steric hindrance, making it difficult for them to penetrate the internal micropores of plant cell walls, which subsequently limits the profound hydrolysis of cellulose [14]. Consequently, a mechanistic opportunity exists for a compound that can simultaneously reduce hydrophobic adsorption at the enzyme–membrane interface and stabilize released enzyme proteins in the aqueous phase—precisely the profile that CAPB’s amphoteric molecular architecture is hypothesized to provide [10,11,12].

Given these properties, CAPB may represent a mechanistically distinct approach to enhance rumen fiber digestion—one that operates not merely by improving wettability, but by modifying the enzyme-substrate interface at the microbial level. This possibility remains largely unexplored, as prior research on surfactant supplementation in ruminant nutrition has focused predominantly on substrate wettability effects, without investigating the spatial redistribution of fibrolytic enzymes between the cell-bound and free liquid-phase fractions of rumen fluid [5,6,7]. Based on the aforementioned background, the present study hypothesizes, as a working hypothesis requiring experimental verification, that addition of an optimal amount of CAPB can reduce the interfacial tension of the rumen fermentation system, thereby potentially promoting the dissociation of fibrolytic enzymes originally attached to the bacteria and substrate surfaces from a “bound state” and triggering their extensive release into the fermentation fluid (transitioning to a “free state”)— a phenomenon we term “enzyme elution”. If this mechanism operates as hypothesized, this state transition would not only overcome the physical steric hindrance of the bacteria, allowing enzyme molecules to infiltrate the micropores of roughage for efficient degradation, but also could ensure that this microscopic improvement in fermentation efficiency ultimately translates into macroscopic enhancements in nutrient digestibility and growth performance in live animals. The supplementation gradient evaluated in the present study (0–3.0% of substrate DM in vitro; 0, 0.5%, 1.0%, and 2.0% of dietary DM in vivo) was selected based on the dose range reported in prior studies of analogous non-ionic surfactants in ruminant systems [5,6,7,15,16], with the upper boundary set conservatively to encompass both the anticipated optimal range and the threshold at which cytotoxic effects have been documented for structurally related surfactants [8,17]. To systematically test this hypothesis, this study employed a dual-line research strategy integrating in vitro mechanistic exploration and in vivo validation. Initially, an in vitro yak rumen fermentation model was utilized to systematically evaluate the effects of varying concentrations of CAPB on fermentation kinetics and the spatial distribution patterns of key digestive enzymes, with the aim of elucidating its micro-regulatory mechanisms and identifying the optimal safe dosage. Subsequently, based on the in vitro screening results, an in vivo growth and apparent digestion trial was conducted to further verify the practical efficacy of CAPB on the productive performance of live yaks. This study intends to provide comprehensive support, encompassing both microscopic mechanisms and macroscopic production data, for the application of amphoteric surfactants in efficient ruminant production.

## 2. Materials and Methods

All animal procedures involved in this study complied with the Animal Welfare Guidelines of China. The experimental protocol was reviewed and approved by the Animal Ethics Committee of the Animal Nutrition Institute, Sichuan Agricultural University (Approval No.: 20210628).

### 2.1. Experimental Materials and Basal Diet

The substrate for the in vitro fermentation trial (Experiment 1) and the basal diet for the in vivo feeding trial (Experiment 2) in this study were identical total mixed rations (TMR). This TMR was formulated to meet the nutritional requirements of growing yaks with an average body weight of 135 kg and a target daily weight gain of 600 g/d, according to the nutrient requirements of beef cattle (National Academies of Sciences, Engineering, and Medicine (NASEM), 2016), using rice straw as the primary roughage source [18]. The ingredient composition and conventional nutrient levels of the basal diet are detailed in Table 1.

The feed additive used in the trials was CAPB, provided by Zhengzhou Sansan Chemical Co., Ltd. (Zhengzhou, China). The product appears as a colorless or light-yellow liquid, with the molecular formula C_19_H_38_N_2_O_3_ and the Chemical Abstracts Service registry number 61789-40-0 (86438-79-1). The active ingredient content of this reagent is 30% ± 2%, with a pH value ranging from 6.0 to 7.0. The supplementation doses of CAPB for all treatment groups in the trials were expressed as the percentage of pure active ingredient (a.i.) relative to the substrate or dietary dry matter (DM). All doses were accurately back-calculated from the product solution (active ingredient content: 30% ± 2%) to the required volume for each treatment, ensuring that the stated concentrations (e.g., 0.5%, 1.0%) reflect the a.i. content on a DM basis throughout this study.

### 2.2. Experiment 1: In Vitro Rumen Fermentation

Three adult male yaks in good health and comparable body condition scores were designated as rumen fluid donors throughout the study. To maintain microbial community consistency between experiments, donor animals received the same TMR as that used in Experiment 2 for the duration of the trial. Rumen fluid was withdrawn via an oral stomach tube at approximately 07:00, prior to the morning meal. The first 200 mL collected was discarded to minimize salivary contamination. Subsequently, a total of approximately 1.5 L of rumen fluid (about 500 mL per yak) was collected and pooled into a single mixed inoculum. The pooled fluid was promptly sealed in pre-heated vacuum flasks maintained at 39 °C under a continuous CO_2_ atmosphere and conveyed to the laboratory without delay. Upon arrival, the fluid was strained through four layers of sterile cheesecloth; the resulting filtrate was kept under CO_2_ purging in a 39 °C water bath until use.

The artificial rumen buffer solution was prepared according to a previously described method [19], with the specific composition detailed in Table 2. The buffer solution and the pre-treated rumen fluid were mixed at a volume ratio of 2:1 (*v*/*v*) in a 39 °C water bath with continuous CO_2_ flushing until the pH stabilized and the resazurin indicator turned colorless, ensuring a strictly anaerobic and reduced environment, thereby formulating the mixed incubation medium. Substrate portions of 300 mg were enclosed in heat-sealed nylon filter bags (pore size 42 μm) and placed at the base of individual glass syringes. Each syringe received 30 mL of the incubation medium (comprising 20 mL of buffer solution and 10 mL of rumen fluid) delivered by pipette; air pockets were expelled thoroughly before the outlet was clamped shut. The loaded syringes were secured in an upright orientation in a shaking water bath (SHZ-82, Changzhou Guohua, Changzhou, China) set at 39 °C for anaerobic fermentation.

This experiment employed a randomized block design and was divided into two independent incubation batches. A total of eight groups were established, including seven CAPB treatment groups with different supplementation levels (0%, 0.5%, 1.0%, 1.5%, 2.0%, 2.5%, and 3.0% of the substrate DM, respectively) and one blank control group containing only the mixed incubation medium. Each CAPB treatment group had six replicate fermentation tubes, while the blank control group had two replicates. In each independent incubation batch, half of the replicates for each treatment group were evenly distributed and randomly assigned to three thermostatic shaking incubators. Sampling was conducted in parallel at six time points: 2, 4, 8, 12, 24, and 48 h.

#### 2.2.1. Sample Collection (Experiment 1)

At each designated incubation time point (2, 4, 8, 12, 24, and 48 h), the piston scale of each glass syringe was immediately and accurately read and recorded. To prevent the piston from falling off due to volume overload, when the piston scale exceeded 80 mL during incubation, the reading was accurately recorded, the piston was slowly pushed to exhaust the gas back to the initial scale (30 mL), and incubation continued. The cumulative apparent gas production at a specific time point was the sum of all exhaust readings at and prior to that time.

After reading the gas production, sampling was performed. The syringe sealing clamp was opened, and the mixed fermentation fluid was transferred into sterile centrifuge tubes. First, the pH of the fermentation fluid was directly measured using a high-precision portable pH meter (DLX-PH-05-2, Delixi Electric, Wenzhou, China) calibrated with standard buffer solutions. Subsequently, the majority of the fermentation fluid was aliquoted into cryovials, snap-frozen in liquid nitrogen immediately, and transferred to a −80 °C ultra-low temperature freezer for long-term storage, which was used for the subsequent determination of volatile fatty acids (VFA) and ammonia nitrogen (NH_3_-N). Simultaneously, another 5 mL of fully mixed fresh fermentation fluid was accurately pipetted and immediately used for the real-time separation and extraction of extracellular and intracellular digestive enzymes. The remaining fermentation residues in the nylon bags were repeatedly washed with distilled water until the eluate became clear, and then dried to a constant weight in a 65 °C oven for the subsequent determination of DM and fiber content.

#### 2.2.2. Determination of Gas Production and Calculation of Kinetic Parameters

The actual gas production at a specific time point = apparent gas production of the treatment tube at that time point − gas production of the blank tube at that time point. The gas production kinetic parameters were fitted using the exponential model GP = *a* + *b* (1 − e^−*c*t^) proposed by Ørskov and McDonald [20] where GP is the cumulative gas production at time t (mL), *a* is the gas production from the rapidly degradable fraction (mL), *b* is the gas production from the slowly degradable fraction (potentially degradable fraction) (mL), *c* is the gas production rate constant of the slowly degradable fraction (h^−1^), *t* is the in vitro fermentation time (h), and *a* + *b* is the potential maximum gas production (mL). The extraction of each kinetic parameter in this model was performed by iterative fitting calculations using the non-linear regression procedure in SAS 9.4 statistical software.

#### 2.2.3. Determination of Rumen Fermentation Parameters and Enzyme Activities

Ammonia nitrogen concentration assay: The thawed fermentation fluid was centrifuged at 10,000× *g* for 15 min at 4 °C, and the supernatant was collected for determination. The classic phenol-sodium hypochlorite colorimetric method was employed, and the absorbance was measured at a wavelength of 630 nm using a microplate reader (Multiskan FC, Thermo Fisher Scientific, Waltham, MA, USA); the NH_3_-N concentration was calculated via a standard curve [21].

Microbial protein (MCP) concentration assay: The thawed fermentation fluid was first centrifuged at a low speed of 500× *g* for 5 min at 4 °C to remove feed residues and protozoa. The supernatant was carefully collected and centrifuged again at a high speed of 10,000× *g* for 15 min at 4 °C to collect the microbial cell pellet at the bottom. After resuspending and fully lysing the pellet with a 0.25 mol/L NaOH solution, quantitative determination was performed using a bicinchoninic acid total protein quantitative assay kit (Nanjing Jiancheng Bioengineering Institute, Nanjing, China), with specific operations strictly following the kit instructions.

VFA assay: Quantitative determination was conducted using a gas chromatograph (Agilent 8890, Agilent Technologies, Santa Clara, CA, USA) equipped with a flame ionization detector. A polar capillary column (DB-FFAP, 30 m × 0.25 mm × 0.25 μm) was used. After pre-treatment, the fermentation fluid was mixed with a 25% metaphosphoric acid solution containing crotonic acid as an internal standard at a ratio of 4:1, and centrifuged at 10,000× *g* for 10 min at 4 °C to precipitate proteins. The supernatant was filtered through a 0.22 μm microporous membrane before injection. Gas chromatography conditions: inlet temperature 220 °C, detector temperature 250 °C, high-purity nitrogen as the carrier gas, and separation was run in a programmed temperature mode (specific temperature program: initial column temperature 110 °C, held for 3 min; then increased to 180 °C at a rate of 10 °C/min, and held for 3 min).

Digestive enzyme activities assay: The immediate separation of intracellular and extracellular enzymes was performed on the fresh fermentation mixture after 24 h of in vitro fermentation. Exactly 5 mL of fresh fermentation fluid was pipetted and centrifuged at 10,000× *g* for 15 min at 4 °C. The supernatant was collected and stored in an ice bath as the test solution for extracellular free enzymes. The resulting pellet was resuspended with 5 mL of pre-cooled phosphate-buffered saline (pH 6.8), placed in an ice-water bath, and subjected to cell disruption using an ultrasonic cell disruptor (JY92-IIN, Scientz Biotechnology, Ningbo, China) (ultrasonic power 200 W, ultrasonication for 3 s, interval for 10 s, repeated 30 times). After disruption, it was centrifuged again at 10,000× *g* for 10 min at 4 °C, and the supernatant was collected as the test solution for intracellular bound enzymes. The activities of carboxymethyl cellulase (CMCase), xylanase, α-amylase, and total protease in this experiment were all determined using commercial micro-biochemical assay kits (Shanghai Enzyme-linked Biotechnology Co., Ltd., Shanghai, China) combined with colorimetric analysis using a full-wavelength microplate reader (SpectraMax M2, Molecular Devices, San Jose, CA, USA). To simulate the physiological environment of the rumen, the incubation temperature for all enzymatic hydrolysis reactions was uniformly set at 39 °C. The specific assay principles and methods were as follows: (1) CMCase activity: The 3,5-dinitrosalicylic acid (DNS) colorimetric method was utilized. Using sodium carboxymethyl cellulose as the substrate, the extracted enzyme solution catalyzed substrate degradation to produce reducing sugars, which reacted with the DNS reagent in a boiling water bath to develop color; the absorbance was measured at 540 nm. (2) Xylanase activity: A neutral xylanase kit was employed. The enzyme solution catalyzed the degradation of xylan in a pH 6.0 buffer system to produce reducing oligosaccharides and monosaccharides; after color development by reaction with the DNS reagent, the absorbance was measured at 540 nm. (3) α-Amylase activity: The DNS colorimetric method was used. The enzyme solution was first heated at 70 °C for 15 min to specifically inactivate β-amylase, followed by the addition of the starch substrate for reaction; the absorbance of the generated reducing sugars was measured at 540 nm. (4) Total protease activity: Natural azocasein was used as the substrate. The enzyme solution catalyzed the hydrolysis of casein; after the reaction was terminated, the unreacted macromolecular substrate was precipitated with trichloroacetic acid. The supernatant was centrifuged, and the characteristic light absorption of the free azo-peptides at 366 nm was measured to calculate the total protease activity. The calculation formula for total enzyme activity was as follows: Total enzyme activity = Extracellular free enzyme activity + Intracellular bound enzyme activity.

### 2.3. Experiment 2: In Vivo Growth Performance and Apparent Digestibility Trial

This experiment was conducted at the teaching and research base of the Animal Nutrition Institute, Sichuan Agricultural University (30.3° N, 103.0° E; altitude approximately 600 m; the trial was conducted from 13 June to 1 August 2024, during which the average ambient temperature ranged from 21 to 28 °C and average monthly precipitation was approximately 120 mm). Twenty-four healthy male yaks of similar age (approximately 24 months), with good body condition and similar initial body weights (131.2 ± 8.4 kg), were selected as experimental animals. The experiment employed a completely randomized design, where the yaks were randomly assigned to four treatment groups with six replicates per group. Based on the dose screening results from the previous in vitro fermentation trial, four CAPB supplementation levels were established: 0 (control group), 0.5%, 1.0%, and 2.0% (on a dietary DM basis), representing the optimal range identified in Experiment 1, an intermediate suboptimal dose, and an upper-bound dose for safety boundary characterization, respectively. The detailed literature basis for these dose ranges is described in the Introduction (Section 1, third paragraph).

The basal diet for the experiment was formulated according to the nutrient requirements for beef cattle (NASEM, 2016) to meet the nutritional needs of growing yaks with an average body weight of 135 kg and a target daily weight gain of 600 g/d [18]. The ingredient composition and nutrient levels of the basal diet were identical to those used in the preliminary in vitro trial. Before the morning feeding each day, the corresponding dose of liquid CAPB was accurately weighed, thoroughly premixed with a small amount of concentrate, and subsequently mixed with the roughage in a TMR mixer to prepare the TMR for feeding. To ensure complete intake of the supplemented CAPB, the CAPB-containing premix was offered first as a small portion at the morning feeding, and the remainder of the diet was provided only after this portion was fully consumed by each animal.

The experimental period lasted for 44 days, including a 14-day adaptation period to eliminate stress and allow the yaks to adapt to the experimental diet and environment, followed by a 30-day formal measurement period. During the experiment, all yaks were housed in individual tie-stalls within a well-ventilated and regularly disinfected standardized barn. Feed was distributed in two meals per day at 07:00 and 19:00. Offered amounts were adjusted daily for each animal based on the previous day’s residuals, targeting ort levels of 5–10% of the offered quantity to confirm voluntary intake. Fresh water was available at all times, and all animals received standard antiparasitic and prophylactic treatments at the start of the trial.

#### 2.3.1. Sample Collection (Experiment 2)

At the beginning and end of the formal measurement period, all experimental yaks were weighed on two consecutive days before the morning feeding. The average values were taken as the initial body weight (IBW) and final body weight, respectively, to calculate the average daily gain (ADG) for the entire formal period. Throughout the formal period, the amount of TMR offered to each yak was accurately recorded daily, and the remaining feed (orts) in the feed bunk was collected and weighed before the morning feeding on the following day. These data were used to calculate the average daily feed intake (ADFI, on a DM basis). Finally, the feed-to-gain ratio was calculated based on the ratio of ADFI to ADG.

The apparent nutrient digestibility was determined during the last 5 days of the formal period (days 26 to 30) using the total feces collection method. To ensure the complete collection of feces and minimize interference with the animals’ natural behavior, the experimental yaks were housed in individual tie-stalls equipped with special leak-proof bedding. Trained staff conducted round-the-clock fecal surveillance during the collection window. Each 24 h sampling cycle ran from the morning feeding at 07:00 to 07:00 the following day. Every defecation event was observed and the voided feces were immediately retrieved in full and deposited into a labelled, sealed container assigned to that individual. The cumulative 24 h collection per animal was weighed in its entirety before the morning feeding the next day. After weighing, the entire day’s feces of each individual yak were thoroughly mixed manually in the plastic bucket. Subsequently, a fecal sample was accurately taken, amounting to 10% of the total weight of the fresh feces for that individual on that day. After sampling, the un-acidified and acidified fecal samples (by adding 10 mL of 10% dilute sulfuric acid solution per 100 g of fresh feces, applied to prevent volatilization loss of ammonia nitrogen during storage and sample handling prior to Kjeldahl crude protein determination, while un-acidified samples were retained for the remaining nutrient analyses for which acidification is unnecessary) collected continuously for 5 days from each yak were pooled individually and stored in sealed containers at −20 °C. These samples were used for the subsequent determination of nutrients such as DM, crude protein (CP), crude ash, neutral detergent fiber (NDF), and acid detergent fiber (ADF).

Simultaneously with the fecal collection, the orts from each yak were accurately collected and weighed daily, and a 10% sample of the fresh weight was taken. The ort samples from each yak over the 5 consecutive days were pooled, thoroughly mixed, and stored at −20 °C for subsequent routine nutritional composition analysis to precisely correct the actual daily nutrient intake.

#### 2.3.2. Chemical Analysis and Calculations (Experiment 1 and Experiment 2)

The in vitro fermentation residues collected in Experiment 1, as well as the TMR samples, ort samples, and un-acidified fecal samples collected in Experiment 2, were all dried in a forced-air oven at 65 °C for 48 h to a constant weight, then ground to pass through a 1 mm sieve for later use. The conventional nutritional components of all samples were determined strictly according to the standard methods of AOAC [22]. Specifically, the determination of DM, crude ash, ether extract (EE), gross energy (GE), NDF, and ADF was performed using the aforementioned conventional oven-dried samples (including un-acidified fecal samples); whereas the determination of CP exclusively utilized the acidified fecal samples and was measured directly using the Kjeldahl nitrogen determination method. Furthermore, DM and crude ash were determined using the 105 °C oven drying method and the 550 °C muffle furnace ignition method, respectively; EE was determined using the Soxhlet extraction method; and GE was measured using an oxygen bomb calorimeter (6400, Parr Instrument Company, Moline, IL, USA). The contents of NDF and ADF were determined using the filter bag technique according to the method of Van Soest et al. [23].

Based on the aforementioned chemical analysis results, the in vitro degradation rate of nutrients was calculated using the formula: In vitro degradation rate (%) = [(Weight of the nutrient in the substrate before incubation − Weight of the nutrient in the residue after incubation)/Weight of the nutrient in the substrate before incubation] × 100. The in vivo apparent nutrient digestibility was calculated using the formula: Apparent digestibility (%) = [((Amount offered × Nutrient content of feed) − (Amount of orts × Nutrient content of orts) − (Amount of feces × Nutrient content of feces))/((Amount offered × Nutrient content of feed) − (Amount of orts × Nutrient content of orts))] × 100.

### 2.4. Statistical Analysis

The raw experimental data were initially organized using Microsoft Excel (2019) and subsequently analyzed using SPSS 29.0 statistical software (IBM Corp., Armonk, NY, USA). Prior to all inferential analyses, the assumptions of normality of residuals and homogeneity of variance were verified using the Shapiro–Wilk test and Levene’s test, respectively, and potential outliers were screened by inspection of standardized residuals; all parameters reported in this study satisfied these assumptions, supporting the validity of the parametric tests employed.

For the in vitro fermentation trial data, an analysis of variance was performed using a general linear model (GLM), in which the CAPB supplementation level was designated as a fixed effect, and the independent incubation batches were included as a random block effect for correction. In this experiment, the individual fermentation tube was treated as the experimental unit (*n* = 6 per treatment group). Because each fermentation tube was independently sampled and terminated at each designated time point rather than measured repeatedly over time, each observation at each time point was treated as an independent measurement, and no correction for temporal autocorrelation was applied. Additionally, orthogonal polynomial contrasts were utilized to evaluate the linear and quadratic response patterns of various parameters to the equally spaced CAPB supplementation levels. The non-linear regression fitting of the gas production kinetic parameters (a, b, c) was performed using the NLIN procedure in SAS 9.4 (SAS Institute Inc., Cary, NC, USA), as described in Section 2.2.2.

For the in vivo trial data, analysis was conducted using a GLM for a completely randomized design, wherein the IBW was included in the model as a covariate for correction when analyzing growth performance indicators such as ADG. The individual animal was treated as the experimental unit (*n* = 6 per treatment group). Considering that the CAPB supplementation gradient in the in vivo trial (0, 0.5%, 1.0%, 2.0%) was unequally spaced, a curve estimation procedure was employed with the actual supplementation dose as a continuous variable to thoroughly analyze the linear and quadratic response effects. When the main effect of the model was significant, multiple comparisons were performed using Duncan’s multiple range test. Duncan’s test was selected over more conservative methods (e.g., Tukey’s HSD or Bonferroni correction) because the present study involved a relatively small number of treatment groups (7 groups in vitro with *n* = 6 replicates per CAPB treatment group; 4 groups in vivo with *n* = 6 animals per group), a context in which Duncan’s test has been widely applied and accepted in the ruminant nutrition literature and provides adequate control of experiment-wise error without excessive loss of statistical power. All data results were expressed as mean ± standard error of the mean (SEM). A *p* < 0.05 was considered to indicate a statistically significant difference, while 0.05 < *p* < 0.10 was considered to represent a significant statistical trend.

## 3. Results

### 3.1. pH of Fermentation Fluid

Under the present experimental conditions, different supplementation levels of CAPB had no significant effect on the pH of the in vitro rumen fermentation fluid in yaks (*p* > 0.05) (Table 3). Neither the main effect analysis nor the linear and quadratic regression analyses revealed any significant differences among the treatment groups at any time point. Throughout the entire fermentation process (2 to 48 h), the pH values of the fermentation fluid in all groups fluctuated within a narrow range of 6.39 to 6.73. With prolonged fermentation time, the pH of the fermentation fluid exhibited a gradual decreasing trend, but consistently remained between 6.0 and 7.0. These results indicate that the fermentation environment was relatively stable across all groups, and the supplementation of CAPB did not negatively affect the ruminal acid-base balance. The pH values in all treatment groups were maintained within the normal physiological range suitable for rumen microbial fermentation.

### 3.2. Gas Production and Kinetic Parameters

The supplementation of different levels of CAPB significantly affected the in vitro gas production and kinetic parameters of yak rumen fermentation (Table 4). The main effect analysis revealed significant differences in the cumulative gas production among the groups at 24 and 48 h of incubation (*p* < 0.01). Regarding the fermentation trend, the gas production exhibited a significant quadratic response with increasing CAPB supplementation levels, initially increasing and subsequently decreasing (Quadratic, *p* < 0.01). Specifically, the 48 h cumulative gas production reached its maximum in the 0.5% and 1.0% addition groups (250.61 and 252.67 mL/g DM, respectively), which were significantly higher than those in the 2.5% and 3.0% groups (*p* < 0.05). When the supplementation level exceeded 2.0%, the gas production dropped significantly, with the lowest value recorded in the 3.0% group (201.00 mL/g DM), suggesting that a high concentration of CAPB was associated with a reduction in fermentation parameters under the present experimental conditions.

In terms of gas production kinetic parameters, CAPB had no significant effect on the gas production from the rapidly degradable fraction (*a*) or the gas production rate constant (*c*) (*p* > 0.05). However, the gas production from the slowly degradable fraction (*b*) was significantly affected (*p* < 0.05) and similarly demonstrated a significant quadratic trend (*p* < 0.05). These results suggest that CAPB primarily influences the total gas production by altering the fermentation extent of slowly degradable components, such as fiber, in the diet, and that an optimal supplementation dose (0.5–1.0%) contributes to enhancing the fermentation potential of the substrate.

### 3.3. In Vitro Degradability of Dry Matter and Fiber

Regarding the in vitro dry matter degradability (IVDMD), no significant differences were observed among the treatment groups during the initial stage (2–12 h) and at the end of fermentation (48 h) (*p* > 0.05). However, during the middle stage of fermentation (24 h), the CAPB supplementation level significantly affected IVDMD (*p* < 0.05). The IVDMD in the 0.5% and 1.0% addition groups reached the highest values (39.49% and 39.70%, respectively), which were significantly higher than that in the 3.0% high-dose group (*p* < 0.05). Trend analysis revealed that, although the main effect differences were not significant at certain time points (such as 12 h and 48 h), IVDMD consistently exhibited a significant quadratic trend, initially increasing and subsequently decreasing with the increment of CAPB supplementation level (Quadratic, *p* < 0.05). This indicates that the supplementation of an optimal amount of CAPB (0.5–1.0%) has a potential promotional effect on substrate degradation, whereas a high dose was associated with reduced substrate degradation.

Under the present experimental conditions, the addition of CAPB significantly altered the degradation characteristics of substrate in vitro neutral detergent fiber degradability (IVNDFD), but had no significant effect on in vitro acid detergent fiber degradability (IVADFD). Specifically, IVNDFD was significantly affected by the CAPB supplementation level during the middle stage (24 h) and at the end of fermentation (48 h) (*p* < 0.05). At 24 h of fermentation, IVNDFD in the 0.5% addition group reached a peak (32.78%), which was significantly higher than that in the 3.0% high-dose group (27.29%) (*p* < 0.05), indicating that an optimal amount of CAPB effectively accelerated the fiber degradation process during the vigorous fermentation stage. By 48 h of fermentation, this promotional effect persisted, with IVNDFD in the 0.5–1.0% addition groups remaining significantly higher than that in the 3.0% group (*p* < 0.05). Furthermore, regression analysis showed that IVNDFD exhibited a significant quadratic trend at both 24 and 48 h (Quadratic, *p* < 0.01). In contrast, there were no significant differences in IVADFD among the treatment groups throughout the entire fermentation process (2–48 h) (*p* > 0.05). However, trend analysis indicated that at the end of fermentation (48 h), IVADFD exhibited significant linear and quadratic responses with increasing CAPB supplementation concentrations (*p* < 0.05) (Table 5).

### 3.4. Concentrations of Ammonia Nitrogen and Microbial Protein in Fermentation Fluid

The supplementation of CAPB significantly altered the nitrogen metabolism characteristics in the in vitro rumen fermentation system of yaks, demonstrating a distinct dose-dependent effect (Table 6). Regarding NH_3_-N, significant differences were observed among the groups at 12 and 24 h of fermentation (*p* < 0.05). Particularly during the vigorous fermentation stage (24 h), as the CAPB supplementation level increased, the NH_3_-N concentration exhibited a significant quadratic trend, initially decreasing and then increasing (Quadratic, *p* < 0.05). Notably, the NH_3_-N concentration in the 1.0% addition group was the lowest (15.64 mg/dL), which was significantly lower than that of the control group (20.49 mg/dL) and the 3.0% high-dose group (22.81 mg/dL) (*p* < 0.05). By the end of fermentation (48 h), the NH_3_-N concentrations among all groups tended to be uniform, with no significant differences (*p* > 0.05). Regarding MCP, its variation trend showed a high negative correlation with ammonia nitrogen. The MCP production was significantly affected by the CAPB supplementation level at both 24 and 48 h of fermentation (*p* < 0.05). At 24 h, the MCP concentrations in the 0.5% and 1.0% addition groups were significantly higher than that in the 3.0% high-dose group (*p* < 0.05), reaching peak values (3.01 and 3.03 mg/mL, respectively). The regression analysis revealed that MCP exhibited a significant quadratic response to the supplementation level at both 24 and 48 h (Quadratic, *p* < 0.05).

### 3.5. Concentration and Composition of Volatile Fatty Acids in Fermentation Fluid

The effect of CAPB on the in vitro rumen fermentation VFA of yaks was primarily reflected in the enhancement of fermentation production and the utilization of intermediate metabolites, without significantly altering the main fermentation type (Table 7). Regarding total volatile fatty acids (TVFA), the supplementation of CAPB exerted a significant promotional effect during the vigorous fermentation stage (24 h). The TVFA concentration showed a significant quadratic trend with the addition level (Quadratic, *p* < 0.01), where the TVFA productions in the 0.5% and 1.0% addition groups were the highest (79.42 and 81.20 mmol/L, respectively), significantly higher than those in the 2.5% and 3.0% high-dose groups (*p* < 0.05). At the initial fermentation stage (12 h) and the end of fermentation (48 h), the TVFA concentrations did not differ significantly among the groups (*p* > 0.05). In terms of VFA composition, the molar proportions of acetate, propionate, and butyrate, as well as the acetate to propionate ratio (A/P), were not significantly affected by the CAPB supplementation level at any time point (*p* > 0.05), indicating that CAPB functions primarily by enhancing fermentation intensity rather than shifting fermentation pathways. However, the proportions of branched-chain volatile fatty acids (isobutyrate and isovalerate) and valerate presented a significant quadratic variation at 24 h (*p* < 0.05), with lower values observed in the 0.5% to 1.0% addition groups. In summary, the optimal supplementation of CAPB (0.5–1.0%) significantly increased the TVFA production during the middle stage of fermentation while maintaining a stable rumen fermentation pattern (stable A/P).

### 3.6. Digestive Enzyme Activities and Their Spatial Distribution Characteristics

Under the present experimental conditions, the supplementation of different concentrations of CAPB significantly altered the spatial distribution characteristics of fibrolytic enzymes in the fermentation fluid, but had no significant effect on the degrading enzymes of non-structural carbohydrates and proteins (Table 8). Regarding fibrolytic enzymes, the addition of CAPB profoundly promoted the release of CMCase and xylanase into the free liquid phase. For both CMCase and xylanase, their extracellular free enzyme activities exhibited a significant quadratic trend, initially increasing and subsequently decreasing with the increment of CAPB supplementation concentration (*p* < 0.01), both reaching their peaks in the 1.0% addition group (24.85 and 52.80 U/mL, respectively), which were significantly greater than those in the control group and other high-dose groups (*p* < 0.01). Correspondingly, the intracellular (bound) enzyme activities of these two enzymes showed a significant decrease in the 0.5–1.0% supplementation range (*p* < 0.05). In terms of total enzyme activity, CAPB significantly increased the total production of CMCase in the system (*p* < 0.01), whereas the total activity of xylanase, although reaching its highest numerical value at 1.0%, showed no significant differences among the groups (*p* > 0.05). Regarding α-amylase and protease, at 24 h of fermentation, the extracellular, intracellular, and total enzyme activities among the groups were not significantly affected by the CAPB supplementation level (*p* > 0.05). Neither the main effect analysis nor the trend regression analysis revealed any regular patterns, with these indices merely presenting natural, irregular fluctuations.

### 3.7. Growth Performance

The supplementation of different doses of CAPB in the basal diet had no significant effects on the IBW, final body weight, and ADFI of the experimental yaks (*p* > 0.05) (Table 9). The ADFI among the treatment groups remained stable, fluctuating within the narrow range of 3.42 to 3.60 kg. Regarding weight gain performance, dietary CAPB supplementation significantly affected the ADG of yaks (*p* = 0.027). The multiple comparison results demonstrated that the ADG of yaks in the 0.5% CAPB group was the highest, reaching 611.11 g, which was significantly higher than that of the control group (506.67 g) (*p* < 0.05). However, when the CAPB supplementation dose was further increased, the ADG in the 1.0% and 2.0% groups declined (536.67 and 527.78 g, respectively), showing no significant differences compared with the control group (*p* > 0.05). Furthermore, the ADG did not exhibit any statistically significant linear or quadratic responses with the increasing CAPB supplementation dose (*p* > 0.05). In terms of feed conversion efficiency, the feed-to-gain (F/G) ratio of yaks in all treatment groups did not reach a statistically significant difference level, but presented a strong decreasing trend influenced by the CAPB effect (*p* = 0.088). In absolute terms, the F/G ratio in the 0.5% CAPB group dropped to the lowest level (5.89), representing a numerical improvement of approximately 13.6% compared with the control group (6.82). Similar to the pattern of ADG, the F/G ratio also did not demonstrate any significant linear or quadratic response characteristics (*p* > 0.05). It should be noted that, given the absence of significant linear or quadratic dose–response trends in ADG and the tendency-level significance of the F/G ratio (*p* = 0.088), conclusions regarding optimal supplementation levels and productive efficiency should be interpreted with appropriate caution as indicative findings under the present experimental conditions (*n* = 6 per group, 30-day formal measurement period).

### 3.8. Apparent Total Tract Digestibility

The supplementation of different levels of CAPB in the diet had no significant effects on the apparent digestibilities of DM, organic matter, CP, GE, calcium, and phosphorus in yaks (*p* > 0.05) (Table 10). In terms of fiber and lipid metabolism, the addition of CAPB demonstrated a significant treatment effect. Compared with the control group, the 0.5% CAPB group significantly increased the apparent digestibilities of NDF and EE (*p* < 0.05). For ADF, the 0.5% group reached the highest numerical value (42.01%) and presented a tendency toward improvement compared with the control group (*p* = 0.060).

## 4. Discussion

In the utilization of roughage by ruminants, the dynamic changes in substrate degradation rates and fermentation parameters are the most crucial macroscopic indicators for evaluating fermentation efficiency [24,25]. High-roughage diets, typified by rice straw, severely restrict the initial attachment of rumen microorganisms due to their complex lignocellulosic matrix, high-crystallinity cellulose, and the hydrophobic barrier of lignin [14]. The in vitro fermentation results of the present study demonstrated that the addition of an optimal amount (0.5–1.0%) of CAPB not only significantly enhanced cumulative gas production but also maximized the degradation rates of IVDMD and IVNDFD. More importantly, this micro-fermentation trend was validated at the live animal level: in vivo feeding of 0.5% CAPB significantly improved the apparent digestibility of NDF in yaks. This mutual validation between in vitro and in vivo data not only confirms the reliability of the in vitro model for dose screening but also indicates that CAPB exhibits high stability in the authentic rumen microecology. During the in vitro trial, the pH of the fermentation fluid remained within the normal physiological range (6.39–6.73), indicating the excellent biocompatibility of CAPB.

Compared with traditional non-ionic surfactants (e.g., Tween 80), CAPB exhibited a unique dosage advantage. Previous studies have indicated that the supplementation of other non-ionic surfactants (such as alkyl polyglycosides or Tween 80) can improve roughage degradation or animal digestibility [15,16]; however, significant effects in in vitro fermentation are typically only observed at high supplementation levels of 2.0% or greater. This is likely because non-ionic compounds lack electrical charges, resulting in a weak binding affinity to the substrate within the complex rumen fluid. In contrast, as an amphoteric surfactant, the CAPB molecule simultaneously possesses quaternary ammonium cationic and carboxylic anionic groups [26]. We hypothesize that this unique electrostatic property enables it to delicately mediate the interaction between negatively charged rumen bacteria and hydrophobic plant cell walls, thereby potentially reducing the solid–liquid interfacial tension. This inferred improvement in substrate wettability would, in principle, provide the fibrolytic bacterial consortia with more abundant and expansive primary attachment sites, which is the prerequisite for triggering subsequent highly efficient degradation.

Elucidating the underlying mechanisms responsible for the improved apparent fiber degradation rate represents the core scientific contribution of this study. During natural rumen fermentation, key fibrolytic bacteria (e.g., *Ruminococcus albus* and *Fibrobacter succinogenes*) primarily anchor tightly to the surface of feed particles via peripheral “cellulosomes” or bound enzymes to perform “contact substrate hydrolysis” [27]. This mechanism directly leads to profound spatial heterogeneity in the community distribution and digestive enzyme activities between the solid-phase and liquid-phase microecology within the rumen [28,29]. Nevertheless, intact bacterial cells are voluminous and generally incapable of penetrating the micrometer- or even nanometer-scale pores inside plant cell walls. This severe physical steric hindrance is considered the greatest bottleneck restricting the profound deconstruction of lignocellulose [30].

By determining the micro-enzymatic spatial distribution, this study documented, for the first time in the yak rumen fermentation system, the intense “enzyme elution” phenomenon apparently associated with CAPB. The data revealed that the introduction of 1.0% CAPB led to a sharp decline in the activities of intracellular and tightly bound CMCase and xylanase, while their activities in the free liquid phase exhibited a significant increase. This indicates that CAPB acts not merely as a substrate wetting agent, but rather as a mild “biological eluent”. Owing to its characteristic of stabilizing protein conformations, CAPB may attenuate the non-specific hydrophobic adsorption between fibrolytic enzymes and bacterial outer membranes or substrate surfaces without causing enzyme denaturation and inactivation, potentially facilitating the release of bound enzyme molecules attached to the cell periphery and substrate surfaces into the fermentation fluid. These liberated enzymes may overcome the physical steric hindrance, enabling them to freely diffuse and deeply penetrate into the microscopic pores of roughage for highly efficient hydrolysis [31]. Such surfactant-mediated non-specific promotion of enzyme release has also been observed in the enzyme secretion systems of other microorganisms (e.g., *Burkholderia glumae*) [32]. This shift in the spatial distribution of enzymes provides mechanistic support consistent with the macroscopic improvement in fiber degradation rates observed in this study [33]. It is important to acknowledge, however, that the concurrent increase in MCP production at optimal CAPB doses (Table 6) raises a plausible alternative interpretation: enhanced microbial biomass could independently drive greater extracellular enzyme secretion through normal biosynthetic pathways, producing the same analytical outcome as physical elution. While the present dataset cannot formally exclude this contribution, the enzyme-class specificity of the redistribution—significant for fibrolytic CMCase and xylanase but absent for α-amylase and protease produced by the same microbial community—argues against a non-specific biomass-driven effect and supports physical elution as the primary mechanism. This interpretive ambiguity is acknowledged as a limitation of the present study, and future proteomic approaches are recommended to formally distinguish these two contributions.

The accelerated decomposition of carbohydrate substrates inevitably triggers a systematic remodeling of fermentation end-products and energy metabolism. In the in vitro fermentation system, the highly efficient degradation of cellulose and hemicellulose was associated with a significant increase in TVFA production. VFAs are the primary energy precursors for ruminants, and the elevation in their production means that a more abundant ATP supply is provided for the synthesis of microbial proteins [34]. In the rumen ecosystem, NH_3_-N is the main, or even the sole, nitrogen source for the vast majority of rumen microorganisms (especially fibrolytic bacteria) to synthesize MCP [35,36,37]. The present study found that the supplementation of an optimal amount of CAPB significantly decreased the concentration of free NH_3_-N in the fermentation fluid, accompanied by a significant increase in MCP production. This inverse relationship between NH_3_-N and MCP is consistent with the energy–nitrogen synchronization model [38]. Driven by an abundant carbon skeleton, rumen microorganisms may have accelerated the assimilation of ammonia, thereby likely reducing nitrogen volatilization losses and potentially enhancing the biological utilization value of crude protein.

Furthermore, the in vivo validation trial revealed a notable finding: the apparent digestibility of EE in yaks of the 0.5% CAPB group increased significantly by 10.8% relative to controls. In ruminants, extensive microbial biohydrogenation of dietary fatty acids in the rumen typically results in a predominance of saturated fatty acids reaching the small intestine, which limits overall lipid digestibility [39]. We tentatively propose, as one speculative mechanistic explanation, that a fraction of dietary CAPB may survive ruminal passage and enter the proximal small intestine with the digesta, where its amphiphilic molecular structure could theoretically act in concert with endogenous bile acids to reduce lipid droplet particle size and expand the interfacial area accessible to pancreatic lipase, thereby facilitating intestinal lipid absorption [40]. However, it must be emphasized that post-ruminal CAPB concentrations, small intestinal digesta composition, and in vivo lipid droplet size were not measured in the present study, and this hypothesis therefore remains speculative pending direct experimental verification through duodenal cannulation or isotopic tracer approaches. An alternative explanation, more directly consistent with the present data, is that CAPB may have improved the liberation of feed-encapsulated lipids from the plant cell wall matrix within the rumen itself by reducing surface hydrophobicity, thereby increasing their accessibility to ruminal lipases and microbial esterases prior to intestinal absorption—a mechanism that would not require post-ruminal CAPB bioavailability.

The associated improvement in feed conversion efficiency and growth performance represents the macroscopic production outcomes observed in this study under the present experimental conditions. In the in vivo trial, under the premise of maintaining a stable ADFI, the ADG of yaks in the 0.5% CAPB group significantly increased, and the F/G ratio exhibited a strong decreasing trend. Analyzed using the theory of the ruminant net energy system, this improvement in production benefits stems from the repartitioning of dietary nutrients. Given that the maintenance net energy requirement of yaks is relatively constant, 0.5% CAPB may have enhanced the extraction of fiber and lipid energy from the given diet via the proposed enzyme elution mechanism in the rumen and a putative lipid emulsification effect in the small intestine. Any such extra net energy above maintenance would have been channelled toward retained energy for growth, which is consistent with the observed improvement in feed conversion efficiency macroscopically. It is noteworthy that, despite the significant pairwise difference in ADG between the 0.5% CAPB group and the control (*p* = 0.027), neither the linear nor the quadratic regression of ADG against the supplementation dose reached statistical significance (Linear *p* = 0.780; Quadratic *p* = 0.264). This pattern—wherein a single intermediate dose produces a significant pairwise effect while the overall dose–response regression is non-significant—warrants careful interpretation. We propose that this outcome most likely reflects the combined influence of three factors: (1) the inherent biological variability of live animal production trials, which is substantially greater than that of the controlled in vitro batch-culture system and reduces statistical power for detecting smooth dose–response curves; (2) the moderate per-group sample size (*n* = 6), which limits the precision of the regression estimates; and (3) the compressed effective dose window in the authentic rumen environment, where continuous liquid outflow, salivary buffering, and digesta passage may narrow the range of doses that produce a discernible production response compared with the closed in vitro system. The directional consistency between ADG and the F/G ratio (*p* = 0.088), both numerically optimal at 0.5% CAPB, lends additional support to the biological plausibility of the effect at this dose. These findings should therefore be interpreted as providing preliminary evidence for 0.5% CAPB as the most efficacious dose under the present experimental conditions, while acknowledging that definitive characterization of the dose–response relationship in vivo will require larger-scale trials with greater statistical power.

However, strict dosage control is a critical prerequisite for the application of such regulators. The dose–response analysis in this study revealed a distinct threshold effect: in the in vitro trial, the 3.0% high dose led to a conspicuous decline in gas production and dry matter degradability; in the in vivo trial, the growth advantage in the 2.0% high-dose group also diminished. This aligns with the critical micelle concentration theory of surfactants. When the CAPB concentration exceeds the safety boundary, excessive amphoteric molecules may insert into and disrupt the lipid bilayer of the cell membranes of rumen microorganisms (especially protozoa and sensitive bacteria), potentially leading to the leakage of intracellular contents and destabilization of the microbial ecosystem [17]. Therefore, under the present experimental conditions, 0.5% emerged as a candidate optimal supplementation dose that appears to balance fermentation-promoting efficiency and microecological safety.

Several limitations of the present study should be acknowledged. First, the proposed ‘enzyme elution’ mechanism, while supported by consistent patterns in enzyme spatial distribution data, was not directly demonstrated through microbial attachment dynamics, single-cell enzyme localization techniques, or proteomic characterization of extracellular enzyme pools; it therefore remains a working hypothesis. Second, the in vivo trial was conducted over a 30-day formal measurement period with *n* = 6 animals per group, which limits the statistical power for detecting smooth dose–response relationships and the generalizability of the productive performance findings. Third, the fate of CAPB within the gastrointestinal tract—including its ruminal degradation rate, post-ruminal bioavailability, and potential interactions with intestinal absorptive processes—was not characterized, constraining the mechanistic interpretation of the observed EE digestibility improvement. Future studies employing rumen cannulation, microbial community profiling, and stable isotope tracing are warranted to address these limitations and provide direct mechanistic validation of the proposed effects.

## 5. Conclusions

This study provides the first systematic observation that CAPB, an amphoteric surfactant, is associated with enhanced roughage utilization efficiency in yaks, putatively through the spatial redistribution of fibrolytic enzymes from a cell-bound state to a free liquid-phase state—a phenomenon termed ‘enzyme elution’ that is consistent with the enzyme distribution data presented in Table 8 and that constitutes the primary empirical contribution of this study. An improvement in substrate wettability, a biologically plausible complementary mechanism, was not directly quantified in this study and therefore remains an inferred rather than demonstrated mechanism. Under the present experimental conditions, this spatial redistribution of fibrolytic enzymes was associated with macroscopic improvements in live animals, with 0.5% CAPB significantly increasing NDF and EE apparent digestibility and ADG, and showing a tendency to improve the F/G ratio, all without compromising voluntary feed intake. Doses exceeding the safety threshold (≥2.0% in vivo) negated these benefits, confirming that strict dosage control is essential. These findings identify 0.5% CAPB as a practical and safe candidate rumen regulator under the present experimental conditions, with potential applicability for improving roughage utilization in growing yaks fed high-roughage diets during the dry season in alpine pastoral areas; broader generalization, however, will require larger-scale and longer-duration trials. Future mechanistic verification using rumen cannulation, microbiome profiling, and proteomic approaches is warranted.

## Figures and Tables

**Table 1:** Ingredients and chemical composition of the basal diet (DM basis).

Ingredients	Proportion (%)	Nutrient Levels	Content
Corn	27.07	NE_m_, MJ/kg	5.91
Soybean meal	8.70	NE_g_, MJ/kg	2.77
Wheat bran	4.20	CP, %	9.72
Sodium chloride	1.00	RDP, %	5.54
Calcium carbonate	0.25	RUP, %	4.18
Premix ^a^	0.42	CF, %	24.41
Oat straw	18.36	NDF, %	47.07
Rice straw	40.00	ADF, %	32.77
Total	100	Ca, %	0.28
		P, %	0.22

^a^ The premix provided the following per kg of diets: Vitamin A 4500 IU, Vitamin D_3_ 900 IU, Vitamin E 150 IU, Fe (as ferrous sulfate) 100 mg, Zn (as zinc sulfate) 75 mg, Mn (as manganese sulfate) 55 mg, Cu (as copper sulfate) 10 mg, I (as potassium iodide) 0.6 mg, Se (as sodium selenite) 0.3 mg, Co (as cobalt chloride) 0.15 mg. Note: DM, dry matter; CP, crude protein; RDP, rumen degradable protein; RUP, rumen undegradable protein; CF, crude fiber; NDF, neutral detergent fiber; ADF, acid detergent fiber; NE_m_, net energy for maintenance; NE_g_, net energy for gain. 1 MJ = 0.239 Mcal.

**Table 2:** Composition of the artificial rumen incubation medium.

Solution	Component	Concentration (g/L)
Micro-mineral solution (A)	CaCl_2_·2H_2_O	13.2
	MnCl_2_·4H_2_O	10.0
	CoCl_2_·6H_2_O	1.0
	FeCl_3_·6H_2_O	8.0
Macro-mineral solution (B)	Na_2_HPO_4_ (anhydrous)	5.7
	KH_2_PO_4_	6.2
	MgSO_4_·7H_2_O	0.6
Buffer solution (C)	NH_4_HCO_3_	4.0
	NaHCO_3_	35.0
Resazurin solution (D)	Resazurin	1.0
Reduction solution (E) ^a^	1 mol/L NaOH	4.0 mL
	Na_2_S·9H_2_O	0.625 g
	Distilled water	Up to 100 mL

^a^ The working medium was prepared by mixing distilled water (474 mL), micro-mineral solution (0.12 mL), macro-mineral solution (237 mL), buffer solution (237 mL), resazurin solution (1.22 mL), and reduction solution (47.5 mL).

**Table 3:** Effects of different concentrations of CAPB on pH of in vitro rumen fermentation in yaks (Experiment 1; *n* = 6 per CAPB treatment group).

Time (h)	CAPB Supplementation Level (% of DM)							SEM	*p*-Value		
	0	0.5	1.0	1.5	2.0	2.5	3.0		Treatment	Linear	Quadratic
2	6.70	6.73	6.67	6.73	6.71	6.69	6.69	0.009	0.650	0.551	0.755
4	6.65	6.71	6.70	6.66	6.64	6.65	6.69	0.010	0.326	0.579	0.794
8	6.61	6.64	6.58	6.65	6.63	6.61	6.58	0.012	0.634	0.554	0.496
12	6.52	6.56	6.57	6.49	6.50	6.53	6.54	0.013	0.627	0.746	0.861
24	6.47	6.48	6.42	6.46	6.48	6.45	6.44	0.009	0.503	0.456	0.748
48	6.42	6.39	6.43	6.46	6.45	6.41	6.45	0.007	0.137	0.165	0.298

**Table 4:** Effects of different concentrations of cocamidopropyl betaine (CAPB) on in vitro gas production kinetics and fermentation parameters of yak rumen (Experiment 1; *n* = 6 per CAPB treatment group).

Time (h)	CAPB Supplementation Level (% of DM)							SEM	*p*-Value		
	0	0.5	1.0	1.5	2.0	2.5	3.0		Treatment	Linear	Quadratic
Gas production (mL/g DM)											
2	32.11	30.00	33.28	33.56	32.11	31.72	30.67	0.529	0.561	0.784	0.430
4	48.17	50.72	49.50	46.45	49.33	46.22	45.89	0.777	0.558	0.143	0.283
8	91.06	95.34	93.39	91.11	89.89	88.06	85.67	1.074	0.265	0.021	0.033
12	114.83	119.44	119.61	112.39	118.78	108.89	108.11	1.439	0.109	0.035	0.036
24	172.22 ^a^	183.72 ^a^	181.44 ^a^	169.00 ^ab^	171.33 ^a^	155.67 ^bc^	149.34 ^c^	2.446	<0.01	<0.01	<0.01
48	234.00 ^a^	250.61 ^a^	252.67 ^a^	228.67 ^a^	234.67 ^a^	203.56 ^b^	201.00 ^b^	4.011	<0.01	<0.01	<0.01
Kinetic parameters											
Rapidly degradable fraction (*a*) mL/g DM	10.20	7.63	10.70	11.79	10.22	10.71	10.39	1.060	0.980	0.680	0.887
Slowly degradable fraction (*b*) mL/g DM	256.15 ^abc^	287.18 ^ab^	327.28 ^a^	247.20 ^bc^	265.65 ^abc^	209.40 ^c^	223.33 ^bc^	10.01	0.024	0.022	0.021
Gas production rate (*c*) h^−1^	0.045	0.045	0.041	0.044	0.046	0.053	0.053	0.003	0.866	0.220	0.327
Potential GP (*a + b*) mL/g DM	266.35	294.81	337.99	258.99	275.87	220.11	233.72	10.556	0.051	0.035	0.033

Note: Values within the same row with different lowercase superscripts indicate significant differences (*p* < 0.05), while no letters or the same letters indicate no significant differences (*p* > 0.05). *P*-Linear and *P*-Quadratic, *p*-values for linear and quadratic responses using orthogonal polynomial contrasts, respectively. SEM, standard error of the mean.

**Table 5:** Effects of different concentrations of CAPB on in vitro dry matter (IVDMD), neutral detergent fiber (IVNDFD) and acid detergent fiber (IVADFD) degradability of yak rumen fermentation over time (Experiment 1; *n* = 6 per CAPB treatment group).

Time (h)	CAPB Supplementation Level (% of DM)							SEM	*p*-Value		
	0	0.5	1.0	1.5	2.0	2.5	3.0		Treatment	Linear	Quadratic
IVDMD (%)											
2	4.41	4.29	4.37	4.22	4.32	4.24	4.14	0.058	0.919	0.236	0.497
4	10.70	10.84	11.42	10.25	11.46	11.20	10.81	0.191	0.652	0.698	0.843
8	21.80	23.83	24.59	23.38	23.40	20.65	19.98	0.599	0.313	0.122	0.039
12	30.48	32.22	32.39	31.08	31.45	29.77	28.78	0.403	0.164	0.051	0.017
24	38.92 ^a^	39.49 ^a^	39.70 ^a^	37.17 ^ab^	38.87 ^a^	35.76 ^ab^	33.98 ^b^	0.557	0.031	0.002	0.002
48	43.47	43.71	44.31	42.42	43.04	40.65	39.44	0.559	0.193	0.011	0.016
IVNDFD (%)											
2	1.99	1.83	2.01	2.02	1.69	1.60	1.72	0.074	0.618	0.125	0.295
4	4.77	4.91	5.22	4.90	5.70	4.85	4.63	0.175	0.759	0.985	0.459
8	12.11	12.95	13.47	12.78	11.84	11.47	10.96	0.254	0.092	0.022	0.009
12	19.92	21.47	21.04	19.65	20.08	17.78	17.21	0.459	0.107	0.009	0.010
24	29.78 ^ab^	32.78 ^a^	30.95 ^ab^	32.78 ^a^	29.52 ^ab^	28.26 ^b^	27.29 ^b^	0.547	0.033	0.017	0.005
48	34.61 ^a^	35.39 ^a^	35.51 ^a^	35.19 ^a^	36.73 ^a^	33.65 ^ab^	31.44 ^b^	0.438	0.032	0.054	0.004
IVADFD (%)											
2	0.81	0.85	0.81	0.73	0.87	0.84	0.76	0.027	0.843	0.797	0.960
4	2.69	2.91	2.41	2.89	2.69	2.61	2.69	0.085	0.771	0.770	0.959
8	8.34	8.90	9.33	8.70	9.14	7.67	8.34	0.210	0.416	0.368	0.277
12	13.78	12.92	14.07	14.32	13.19	11.94	13.74	0.353	0.630	0.555	0.830
24	24.41	23.18	21.83	22.77	23.17	22.48	20.65	0.369	0.178	0.026	0.087
48	28.43	31.17	30.72	29.74	29.12	27.39	26.19	0.570	0.203	0.044	0.021

Note: Values within the same row with different lowercase superscripts indicate significant differences (*p* < 0.05), while no letters or the same letters indicate no significant differences (*p* > 0.05). *P*-Linear and *P*-Quadratic, *p*-values for linear and quadratic responses using orthogonal polynomial contrasts, respectively. SEM, standard error of the mean.

**Table 6:** Effects of different concentrations of CAPB on ammonia nitrogen (NH_3_-N) and microbial protein (MCP) concentrations of in vitro yak rumen fermentation over time (Experiment 1; *n* = 6 per CAPB treatment group).

Time (h)	CAPB Supplementation Level (% of DM)							SEM	*p*-Value		
	0	0.5	1.0	1.5	2.0	2.5	3.0		Treatment	Linear	Quadratic
NH_3_-N (mg/dL)											
12	15.77 ^abc^	14.09 ^c^	14.47 ^bc^	16.90 ^ab^	15.31 ^abc^	17.62 ^a^	17.58 ^a^	0.357	0.018	0.006	0.012
24	20.49 ^a^	18.20 ^ab^	15.64 ^b^	21.46 ^a^	21.08 ^a^	22.08 ^a^	22.81 ^a^	0.618	0.017	0.018	0.022
48	21.29	17.48	16.68	20.49	19.20	21.68	21.75	0.610	0.120	0.152	0.076
MCP (mg/mL)											
12	1.98	2.16	2.21	1.92	2.03	1.97	1.86	0.039	0.197	0.098	0.115
24	2.69 ^ab^	3.01 ^a^	3.03 ^a^	2.79 ^ab^	2.44 ^b^	2.57 ^ab^	2.32 ^b^	0.066	0.012	0.004	0.003
48	2.94 ^ab^	3.33 ^a^	3.38 ^a^	2.89 ^ab^	2.93 ^ab^	2.67 ^b^	2.49 ^b^	0.083	0.028	0.006	0.004

Note: Values within the same row with different lowercase superscripts indicate significant differences (*p* < 0.05), while no letters or the same letters indicate no significant differences (*p* > 0.05). *P*-Linear and *P*-Quadratic, *p*-values for linear and quadratic responses using orthogonal polynomial contrasts, respectively. SEM, standard error of the mean.

**Table 7:** Effects of different concentrations of CAPB on volatile fatty acid (VFA) profiles of in vitro yak rumen fermentation over time (Experiment 1; *n* = 6 per CAPB treatment group).

Items	CAPB Supplementation Level (% of DM)							SEM	*p*-Value		
	0	0.5	1.0	1.5	2.0	2.5	3.0		Treatment	Linear	Quadratic
12 h											
TVFA (mmol/L)	45.78	46.09	45.86	44.06	45.82	44.19	43.93	0.634	0.928	0.296	0.575
Acetate (%)	64.85	65.11	65.05	64.93	65.34	64.73	64.38	0.267	0.984	0.615	0.678
Propionate (%)	21.82	21.95	22.03	21.94	21.85	21.76	21.68	0.133	0.995	0.604	0.737
Butyrate (%)	10.48	10.26	10.42	10.53	10.13	10.51	10.84	0.113	0.786	0.423	0.433
Isobutyrate (%)	0.95	0.9	0.92	0.94	0.95	0.98	1.02	0.023	0.865	0.217	0.295
Valerate (%)	1.05	1.13	1.02	1.04	1.05	1.1	1.15	0.024	0.731	0.429	0.394
Isovalerate (%)	0.85	0.65	0.56	0.62	0.68	0.92	0.93	0.046	0.163	0.170	0.019
Acetate/Propionate	2.98	2.97	2.96	2.96	3.00	2.98	2.98	0.018	0.999	0.832	0.973
24 h											
TVFA (mmol/L)	77.63 ^ab^	79.42 ^a^	81.20 ^a^	73.80 ^ab^	75.42 ^ab^	69.09 ^b^	68.23 ^b^	1.260	0.025	0.001	0.002
Acetate (%)	65.53	66.12	66.05	65.87	65.73	65.24	64.93	0.324	0.965	0.399	0.504
Propionate (%)	22.54	22.86	23.07	22.73	22.66	22.37	22.18	0.186	0.915	0.348	0.396
Butyrate (%)	9.06	8.60	8.45	8.88	9.01	9.41	9.61	0.143	0.311	0.054	0.041
Isobutyrate (%)	0.92	0.75	0.72	0.85	0.88	0.95	1.05	0.031	0.079	0.031	0.008
Valerate (%)	1.12	0.95	0.93	1.05	1.08	1.27	1.36	0.046	0.112	0.018	0.008
Isovalerate (%)	0.83	0.72	0.78	0.62	0.64	0.76	0.87	0.027	0.140	0.905	0.033
Acetate/Propionate	2.92	2.89	2.88	2.91	2.91	2.93	2.93	0.028	0.999	0.720	0.876
48 h											
TVFA (mmol/L)	95.51	101.43	103.18	98.57	96.28	92.53	90.19	1.633	0.338	0.075	0.051
Acetate (%)	66.55	66.83	67.12	66.63	66.71	65.26	65.84	0.273	0.602	0.139	0.222
Propionate (%)	22.82	23.19	24.08	23.09	22.93	22.67	22.59	0.214	0.614	0.348	0.309
Butyrate (%)	8.41	7.95	7.02	8.28	8.26	9.74	9.15	0.256	0.119	0.048	0.048
Isobutyrate (%)	0.85	0.83	0.75	0.82	0.84	0.90	0.95	0.023	0.419	0.103	0.063
Valerate (%)	0.85	0.77	0.64	0.75	0.78	0.93	0.89	0.029	0.120	0.160	0.037
Isovalerate (%)	0.52	0.43	0.39	0.43	0.48	0.50	0.58	0.019	0.138	0.146	0.011
Acetate/Propionate	2.93	2.89	2.80	2.89	2.92	2.89	2.93	0.028	0.930	0.780	0.713

Note: Values within the same row with different lowercase superscripts indicate significant differences (*p* < 0.05), while no letters or the same letters indicate no significant differences (*p* > 0.05). *P*-Linear and *P*-Quadratic, *p*-values for linear and quadratic responses using orthogonal polynomial contrasts, respectively. SEM, standard error of the mean.

**Table 8:** Effects of different concentrations of CAPB on digestive enzyme activities of in vitro yak rumen fermentation at 24 h (Experiment 1; *n* = 6 per CAPB treatment group).

Items	CAPB Supplementation Level (% of DM)							SEM	*p*-Value		
	0	0.5	1.0	1.5	2.0	2.5	3.0		Treatment	Linear	Quadratic
CMCase, U/mL											
Extracellular activity	13.24 ^e^	21.65 ^b^	24.85 ^a^	19.35 ^c^	14.63 ^de^	16.18 ^d^	13.11 ^e^	0.683	<0.01	0.022	<0.01
Intracellular activity	19.89 ^a^	15.20 ^c^	14.65 ^c^	16.77 ^bc^	19.19 ^ab^	18.42 ^ab^	18.39 ^ab^	0.415	<0.01	0.270	0.037
Total activity	33.13 ^cd^	36.85 ^ab^	39.50 ^a^	36.12 ^bc^	33.82 ^bcd^	34.60 ^bc^	31.50 ^d^	0.517	<0.01	0.036	<0.01
Xylanase, U/mL											
Extracellular activity	30.14 ^e^	45.30 ^b^	52.80 ^a^	42.15 ^bc^	38.46 ^cd^	33.22 ^de^	35.15 ^de^	1.33	<0.01	0.211	<0.01
Intracellular activity	42.36 ^a^	33.10 ^b^	31.35 ^b^	35.05 ^ab^	35.34 ^ab^	41.98 ^a^	35.85 ^ab^	1.09	0.032	0.887	0.156
Total activity	72.50	78.40	84.15	77.20	73.80	75.20	71.00	1.30	0.117	0.247	0.055
α-Amylase, U/mL											
Extracellular activity	18.10	16.99	18.41	17.19	18.86	16.15	18.22	0.345	0.381	0.862	0.978
Intracellular activity	22.41	23.44	20.54	22.74	20.98	21.88	23.51	0.578	0.783	0.940	0.614
Total activity	40.51	40.44	38.95	39.93	39.84	38.04	41.73	0.663	0.863	0.981	0.654
Protease, U/mL											
Extracellular activity	14.52	15.14	13.99	15.54	13.62	14.67	13.82	0.347	0.759	0.488	0.732
Intracellular activity	15.23	13.75	15.84	14.29	15.65	16.65	15.00	0.375	0.471	0.355	0.655
Total activity	29.75	28.89	29.83	29.84	29.27	31.32	28.82	0.463	0.849	0.820	0.923

Note: One enzyme unit (U) is defined as the amount of enzyme required to catalyze the release of 1 μg of glucose (for CMCase), 1 nmol of reducing sugar (for xylanase), or 1 mg of maltose (for α-amylase) per minute per milliliter of fermentation fluid at 39 °C. For total protease, one enzyme unit (U) is defined as the amount of enzyme required to cause a change of 1.0 in the absorbance of the reaction system at 366 nm per hour per milliliter of fermentation fluid under the assay conditions. Values within the same row with different lowercase superscripts indicate significant differences (*p* < 0.05), while no letters or the same letters indicate no significant differences (*p* > 0.05). P-Linear and P-Quadratic, *p*-values for linear and quadratic responses using orthogonal polynomial contrasts, respectively. SEM, standard error of the mean.

**Table 9:** Effects of different levels of CAPB on the growth performance of yaks (Experiment 2; *n* = 6 per group).

Items	CAPB Supplementation Level (% of DM)				SEM	*p*-Value		
	0	0.5	1.0	2.0		Treatment	Linear	Quadratic
Initial body weight (kg)	131.0	130.6	129.8	133.5	1.721	0.899	0.590	0.753
Final body weight (kg)	146.2	149.0	145.9	149.3	1.752	0.865	0.643	0.890
Average daily gain (g)	506.67 ^b^	611.11 ^a^	536.67 ^b^	527.78 ^b^	13.659	0.027	0.780	0.264
Average daily feed intake (kg)	3.42	3.59	3.51	3.60	0.051	0.608	0.338	0.587
F/G ratio	6.82	5.89	6.59	6.88	0.156	0.088	0.415	0.233

Note: IBW, initial body weight; ADG, average daily gain; ADFI, average daily feed intake; F/G ratio, feed-to-gain ratio; SEM, standard error of the mean. Values within the same row with different lowercase superscripts indicate significant differences (*p* < 0.05), while no letters or the same letters indicate no significant differences (*p* > 0.05). *P*-Linear and *P*-Quadratic, *p*-values for linear and quadratic responses using orthogonal polynomial contrasts, respectively. SEM, standard error of the mean.

**Table 10:** Effects of different levels of CAPB on the apparent total tract digestibility of yaks (Experiment 2; *n* = 6 per group).

Items	CAPB Supplementation Level (% of DM)				SEM	*p*-Value		
	0	0.5	1.0	2.0		Treatment	Linear	Quadratic
Dry matter (%)	56.07	58.86	56.20	56.98	0.759	0.569	0.998	0.928
Organic matter (%)	58.11	60.21	59.00	58.36	0.638	0.682	0.838	0.670
Crude protein (%)	55.45	56.91	56.11	55.17	0.669	0.821	0.701	0.713
Ether extract (%)	61.96 ^b^	68.67 ^a^	65.43 ^ab^	61.20 ^b^	1.079	0.042	0.369	0.053
Gross energy (%)	55.82	57.91	56.45	56.16	0.677	0.735	0.874	0.789
Neutral detergent fiber (%)	44.33 ^b^	49.45 ^a^	46.88 ^ab^	44.68 ^b^	0.685	0.018	0.560	0.051
Acid detergent fiber (%)	37.80	42.01	39.50	38.25	0.618	0.060	0.649	0.168
Calcium (%)	43.02	42.99	42.87	44.26	0.661	0.881	0.488	0.716
Phosphorus (%)	45.12	45.65	45.48	45.53	0.640	0.994	0.875	0.973

Note: Values within the same row with different lowercase superscripts indicate significant differences (*p* < 0.05), while no letters or the same letters indicate no significant differences (*p* > 0.05). *P*-Linear and *P*-Quadratic, *p*-values for linear and quadratic responses using orthogonal polynomial contrasts, respectively. SEM, standard error of the mean.

## Data Availability

The original contributions presented in this study are included in the article. Further inquiries can be directed to the corresponding author.
